# Clinicopathological features, treatment and outcome of Omani patients with localised prostate cancer

**DOI:** 10.1080/2090598X.2020.1781386

**Published:** 2020-07-06

**Authors:** Shiyam Kumar, Ikram A. Burney, Namrata Satyapal, Joseph Kunju, Mohamed Salim Al-Marhoon, Khurrum Mutahir Siddiqui

**Affiliations:** a Unit of Medical Oncology, Yeovil District Hospital, NHS Foundation Trust, Yeovil, UK; b Unit of Medical Oncology, Department of Medicine, Sultan Qaboos University Hospital, Muscat, Oman; c Unit of Radiation Oncology, National Oncology Center, Royal Hospital, Muscat, Oman; d Unit of Urology, Department of Surgery, Sultan Qaboos University Hospital, Muscat, Oman

**Keywords:** Prostate cancer, radiotherapy, LH-releasing hormone, prostatectomy, Arabs, Oman

## Abstract

**Objectives:** To report the outcomes of Omani men diagnosed with localised prostate cancer (PCa), as PCa incidence is increasing in developing countries and there are scarce data regarding clinicopathological features and outcomes of PCa from the Arab world.

**Patients and methods:** All men diagnosed with localised PCa between January 2006 and December 2017, and treated at a university hospital in Oman were included in the study. Data included demographic information, clinical, laboratory, pathological and radiological features at presentation, treatment modalities, and survival outcomes. Patients were followed until April 2019 or until death for disease-free survival (DFS) and overall survival (OS) whichever came first. Survival rates were estimated using the method of Kaplan and Meier. Univariate and multivariate analysis and Cox regression analyses were performed to study factors affecting DFS and OS.

**Results:** Out of 239 men diagnosed with PCa over the study period, only 47 had localised disease (19.7%). The median age was 69 years. The majority (53.2%) had a Gleason score of ≥8 and a median (range) PSA level of 23.71 (range 0.6 – 452.9)ng/mL. In all, 16 patients received radical surgery, 17 received hormonal therapy along with definitive radiotherapy, while 15 were treated either with medical or surgical castration only. After a median follow-up of 43 months, the median DFS was 44.0 months. The median OS was not reached for the entire cohort. The 5- and 10-year OS rates were 84% and 57%, respectively

**Conclusion:** Omani patients with localised PCa present with a high PSA level and a high Gleason score. Potentially curative treatments options, e.g. radical surgery and radiotherapy, are underutilised. The survival outcomes are similar to studies reported internationally.

**Abbreviations:** (P)ADT: (primary) androgen-deprivation therapy; CAPRA: Cancer of the Prostate Risk Assessment; 3D: three-dimensional; DFS: disease-free survival; HDI: Human Development Index; Linacs, linear accelerators; NCCN: National Comprehensive Cancer Network; OS: overall survival; (m)(CR)PC: (metastatic) (castrate-resistant) prostate cancer; RP: radical prostatectomy; (IM)RT: (intensity modulated) radiotherapy; SQUH: Sultan Qaboos University Hospital

## Introduction

Overall the incidence of prostate cancer (PCa) is significantly higher in Western countries compared to other parts of the world, but the incidence of PCa is rising in the developing world as well [1]. PCa incidence is low in the Arab population. For example, the age standardised incidence of PCa in Oman was 10.2 in 2008 as compared to 119.0 in Sweden in the same year [1]. However, over the past few years, the overall cancer incidence has increased in Oman, and in 2015, PCa was the sixth most common cancer overall, and the most common cancer in males [2].

For localised PCa, radical surgery or radiotherapy (RT) along with androgen-deprivation therapy (ADT), are the treatments of choice [3]. On the other hand, for patients with low-risk disease (based on favourable Gleason score, clinical stage, PSA level, and density of tumour in the core biopsy), active surveillance is a valid option and may help to avoid unnecessary treatment-related adverse events without affecting the disease outcome [4]. With the availability of several treatment options, treatment for localised PCa varies across the globe. Patient and physician preferences play a significant role in the choice of treatment [5]. Patients with PCa in developing countries present at advanced disease stage in comparison to developed countries and have a poorer survival. Moreover, patients with PCa in a similar country can have different outcomes for various reasons including access to healthcare facilities, PSA testing for screening, and complexities of social and genetic processes [6,7].

Although, PCa is one of the top 10 cancers in Oman and the incidence is continuously increasing, there are no published data on the patterns of treatment and the disease outcome amongst Omani patients. We had observed that Omani patients present with more aggressive disease as compared to other parts of the world, hence the present study was undertaken [8,9]. In the present study, we report the outcome of Omani patients diagnosed with localised PCa and correlate the outcome with clinicopathological features and treatment. We also compare the outcomes in Oman with regional and international published data.

## Patients and methods

All patients diagnosed with localised PCa, at the Sultan Qaboos University Hospital (SQUH) between January 2006 and December 2017, were included in this study. The SQUH is one of the two major hospitals in the country, located in capital city of Muscat, providing cancer care to patients from all of Oman. Most of the patients were diagnosed and treated at the SQUH. As radiation facilities are only available at the Royal Hospital, Muscat, all patients were referred there to receive radical RT. Tissue blocks were reviewed at the SQUH for patients who were referred from other hospitals.

Electronic records of all patients were reviewed for demographic features (age at the diagnosis, comorbidities, use of medicines for comorbid conditions), clinicopathological features at presentation (PSA level, Gleason score, tumour percentage in the biopsy material, evidence of perineural invasion, and disease stage), treatment received, and survival until either the last date of follow-up or date of death. We also used Cancer of the Prostate Risk Assessment (CAPRA) and National Comprehensive Cancer Network (NCCN) risk stratification scores for correlation with survival. Nadir PSA levels were also checked during the treatment period.

The American Joint Committee on Cancer (AJCC) staging manual (eighth edition) was used to stage the disease [4]. The NCCN risk stratification criteria at the time of diagnosis (very low, low, intermediate, high and very high) [4] and the CAPRA score (0–2, low risk; 3–5, intermediate risk; 6–10, high risk) [10] were used for risk stratification of patients. Standard treatment options for localised PCa were recorded, which included surgery (open or robot-assisted radical prostatectomy [RP]), RT (conformal and intensity modulated [IMRT]), and ADT, which included LHRH agonist with or without antiandrogens. Castrate-resistant PCa (CRPCa) was defined according to Prostate Cancer Working Group 2 criteria [11]. Imaging studies (CT, MRI and bone scan) for disease staging were used as per European Association of Urology Guidance [3]. Patients diagnosed with PCa after or before the date of the study period, patients not treated at the SQUH or those who were lost to follow-up for >2 years, and patients with metastatic disease, were excluded from the analysis.

The Kaplan–Meier method was used to estimate disease-free survival (DFS) and overall survival (OS). DFS was defined as the time period from the date of diagnosis until disease progression and OS was defined as time from diagnosis to death or 30 April 2019. The chi-square test was used to study the association of factors with dichotomous variables, while the log-rank test and Cox regression analysis were used for time to event. A *P* < 0.05 was considered statistically significant. The IBM Statistical Package for the Social Sciences (version 20 for Windows; SPSS Inc., Chicago, IL, USA) was used for statistical analysis. Approval to conduct the study was sought from the institutional medical research ethics committee.

### Prostate cancer treatment evolution in Oman

#### Systemic anti-cancer therapy

The Unit of Medical Oncology was established at the SQUH in 1999. It has been offering systemic cancer therapy including chemotherapy, hormonal therapy, monoclonal antibodies, tyrosine kinase inhibitors and immunotherapy to all patients for all approved indications. All the treatment modalities are made available soon after approval. There is a well-established tumour board meeting in which all cases are discussed.

#### RT

The Radiation Oncology Department at the National Oncology Center (NOC), the Royal Hospital was established in Oman in December 2004. It is a standalone centre for the entire nation offering radiation oncology services to date. The initial set-up included two linear accelerators (Linacs) – Clinac 6EX and 2300 CD (Varian Medical Systems, Palo Alto, CA, USA). IMRT was commissioned in November 2007. Localised PCa was treated with three-dimensional (3D)-conformal RT from December 2004 to December 2007 and then shifted to the IMRT technique. Dose fractionation during the 3D-conformal RT era ranged between 70 and 76 Gy over 35–38 fractions with field reductions for boost used in radical RT and 64–66 Gy in 32–33 fractions for adjuvant cases. With the shift to IMRT a tangible effort was made to move onto mild–moderate hypofractionation and dose escalation to achieve more robust biochemical and local control, as several studies have suggested favourable outcome. At the NOC the fractionation schemes used for IMRT for radical RT for PCa range between 70–75 Gy in 30–33 fractions. Nodal volume irradiation is done in very-high-risk PCa to a dose of 54–56 Gy in 28–30 fractions along with simultaneous integrated boost to the prostate to a dose of 70 Gy. Dose fractionation schemes for adjuvant RT to the prostate bed with IMRT is 65 Gy/28 fractions. In June 2017, the Department replaced the existing Linacs with a new state of art True Beam STx and True Beam with Aria 13.5 platform from Varian Medical Systems. A volumetric modulated arc RT technique was implemented in January 2018, with further hypofractionation vis-à-vis 60 Gy/20 fractions for low- and intermediate-risk PCa and 70 Gy/28 fractions (Radiation Therapy Oncology Group [RTOG] 0415 protocol) for high-\very-high-risk PCa in radical settings was initiated. The Exac-Trac version 5.5, Brain Lab image guidance system (Brainlab, Munich, Germany) has been replaced by cone-beam CT for setup verification.

#### Surgery for PCa

During the study period, the surgical treatment of PCa at the SQUH has left a lot to be desired. All potential candidates for curative treatment were either not offered appropriate curative treatment, e.g. RP, or did not pursue them for the fear of side-effects. Understandably, fear of urinary incontinence and erectile dysfunction are genuine concerns and are related to surgical expertise. Among the patients who underwent RP, only a few were performed locally and the rest referred abroad. Patients usually travel to India or Thailand to undergo RP. Currently, Oman does not have a robotic surgery programme. Since 2017, the SQUH has initiated dedicated urological oncology clinics for PCa and hopefully we will report the results of its impact in the future.

## Results

A total of 239 patients were diagnosed with localised or metastatic PCa during the study period (January 2006 to December 2017). Out of those, 47 patients met the inclusion criteria and had localised PCa at the time of diagnosis (19.7%). This study reports on the presenting features and outcomes of these 47 patients.

### Clinicopathological features

The median (range) age was 69 (48–83) years, all had symptoms of prostatism at presentation (hesitancy, dribbling, difficulty in initiation of urination, nocturia), and the majority had one or more comorbidities (72.3%). TRUS-guided biopsy was the most common diagnostic method (80.9%). More than half of the patients (53.2%) had a Gleason score of ≥8. The median (range) PSA level at the time of diagnosis was 23.71 (range 0.6 – 452.9)ng/mL. More than 50% of core tissue was involved by the disease in 55% of patients. The vast majority of patients (68.1%) had high-risk disease according to the NCCN and CAPRA risk stratifications, and 53.2% had Stage III at the time of diagnosis (Table 1).

**Table 1.** The patients’ clinicopathological characteristics

Characteristic	*N* (%)
Comorbid conditions	
None	13 (27.7)
Hypertension	30 (63.8)
Diabetes	14 (29.8)
Coronary artery disease	9 (19.1)
Statin use	21 (44.7)
Metformin use	7 (14.9)
Diagnostic method	
TRUS	38 (80.9)
TURP	9 (19.1)
Gleason Score	
≤6	6 (12.8)
7	16 (34.0)
8	12 (25.5)
9	10 (21.3)
10	3 (6.4)
Tumour % in biopsy core	
<20	4 (8.5)
20–50	9 (19.1)
>50	26 (55.3%)
Missing	8 (17.0)
NCCN risk group	
Very low risk	1 (2.1)
Low risk	4 (8.5)
Intermediate risk	7 (14.9)
High risk	27 (57.4)
Very high risk	7 (14.9)
Missing	1 (2.1)
CAPRA risk group	
Low risk	3 (6.4)
Intermediate risk	12 (25.5)
High risk	32 (68.1)
Disease Stage	
Stage I	9 (19.1)
Stage II	13 (27.7)
Stage III	25 (53.2)
PSA level, ng/mL	
<10	12 (25.5)
10–20	6 (12.8)
21–50	17 (36.2)
51–100	2 (4.3)
>100	8 (17.0)
Missing	2 (4.3)
Pathological tumour stage	
pT1	2 (4.3)
pT2	6 (12.8)
pT3a	5 (10.6)
pT3b	2 (4.3)

### Patterns of treatment

Of the 47 patients, 17 (36.2%) received hormonal therapy along with definitive RT. Of these 17 patients, 10 had high-risk disease and five were diagnosed with very-high-risk disease, whereas one patient each had very-low and intermediate-risk disease.

In all, 16 of the 47 patients (34.1%) underwent RP. Of these, the majority (53.2%) had extraprostatic tissue invasion (pT3a or pT3b). Because of advanced tumour size, five patients required adjuvant RT with or without hormonal agents after RP (Table 2). Of these 16 patients, nine had high-risk disease, while three had low-risk disease, and four had intermediate-risk disease. Most of the patients received adjuvant LHRH agonists for ≥2 years (Table 2) and 33 (70.2%) patients reported no side-effects associated with LHRH therapy. Two patients each with low- and high-risk disease had biochemical recurrence requiring salvage RT.

**Table 2.** First-line treatment offered to the patients

Characteristic	*N* (%)
First-line treatment	
Radical IMRT + LHRH	17 (36.2)
RP only	11 (23.4)
LHRH Only	10 (21.3)
RP, LHRH and IMRT	3 (6.4)
RP and IMRT	2 (4.3)
Combined androgen blockade	2 (4.3)
Orchidectomy	1 (2.1)
Orchidectomy and IMRT	1 (2.1)
LHRH duration, years	
0.5	3 (6.4)
2	10 (21.3)
2–3	11 (23.4)
Still receiving as first line	5 (10.6)
Time to nadir PSA, months	
<6	18 (38.3)
>6	27 (57.4)
Missing	2 (4.3)

The remaining 15 (32.0%) patients were treated either with LHRH analogues (seven high-risk disease), combined androgen blockade or orchidectomy despite having localised disease, but did not receive either surgery or RT (Table 2).

Time to nadir PSA was <6 months in 18 (38.3%) patients (Table 2).

### DFS

**Figure 1.**
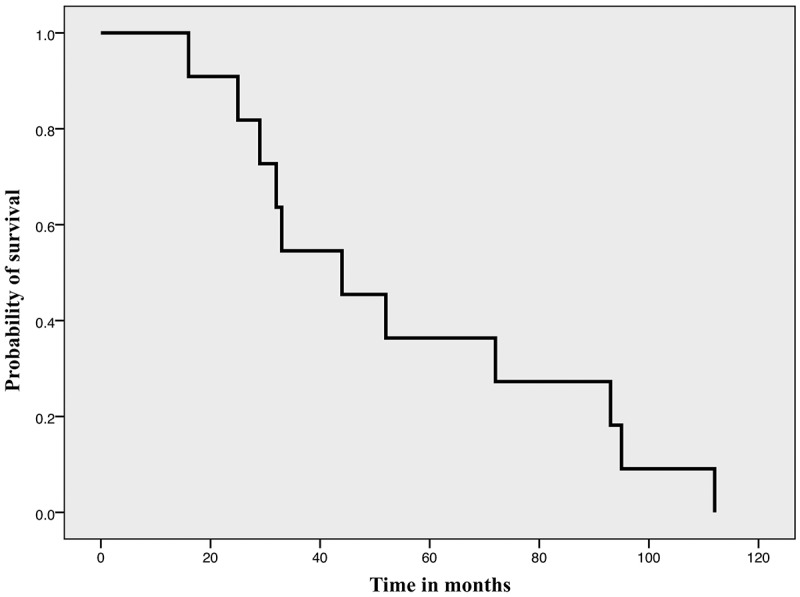
The DFS of all patients

After a median (range) follow-up of 43 (8–132) months, 29 (61.7%) patients were in complete remission, five (10.6%) were still receiving treatment, while 11 (23.4%) had disease relapse. The median DFS was 44.0 months (Figure 1). Age, number of comorbidities, Gleason score, tumour percentage in biopsy core, risk group, pathological tumour size, PSA level at diagnosis, and time to PSA nadir significantly affected the DFS on univariate analysis. None of the factors significantly affected DFS on multivariate analysis.

### Patterns of relapse

Of the 11 patients who had disease progression, five developed metastatic CRPCa (mCRPC), two had local relapse and four had biochemical recurrence only. The median (range) PSA level at the time of relapse was 98.2 (0.7–295.0)ng/mL. All patients with biochemical recurrence were treated with salvage RT along with LHRH and three of these patients are in remission, while one patient developed mCRPCa. Bone and lymph nodes were common sites of metastatic disease in all except one, who also had visceral metastasis. Of the six patients with mCRPCa, three were treated with docetaxel, two with abiraterone, and one was treated by best supportive care due to poor performance status.

### OS

**Figure 2.**
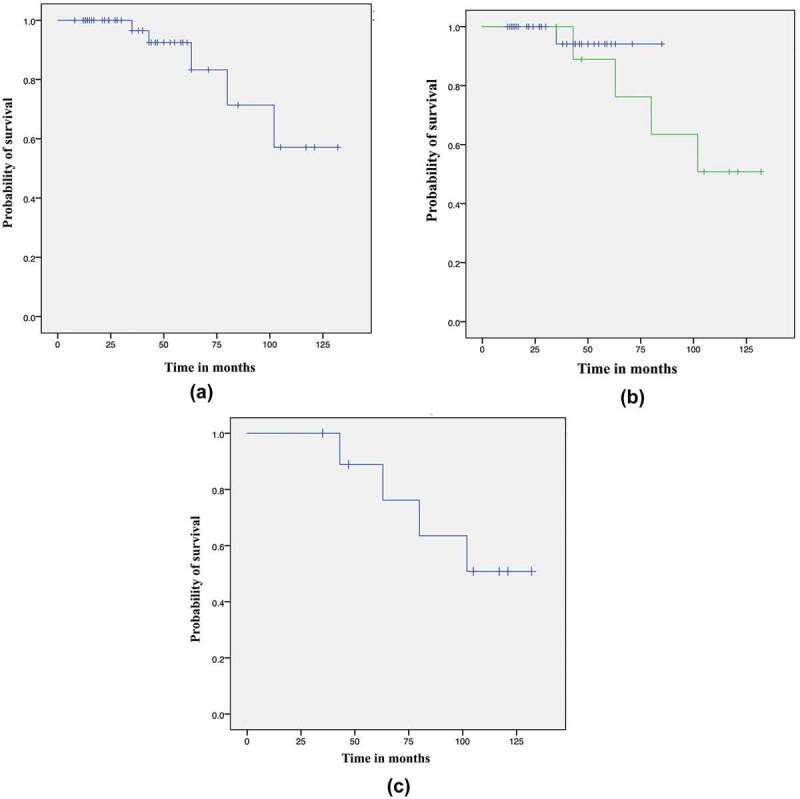
The OS of all patients with localised PCa at the time of diagnosis (a), those with disease in remission (b), and those with disease relapse (c)

At a median (range) follow-up of 43 (8–132) months, the median OS was not reached for the entire cohort, or for patients with or without relapsed disease (Figure 2). The 5- and 10-year OS rates were 84% and 57%, respectively

## Discussion

To the best of our knowledge, this is the first report describing the presenting features and outcomes of PCa from Oman. The majority of men presented with high-risk disease according to the NCCN and CAPRA criteria. Although most of the patients were treated with either surgery or RT, with or without adjuvant LHRH analogues, a small percentage of patients received medical/surgical castration only as the initial treatment.

PCa is the commonest cancer in Omani males and the incidence is continuously rising. Only 30 males were diagnosed with prostate cancer in 2003; the incidence has increased by almost three-fold in 2015 to 83 [2]. This rise could be attributed to several factors, including the ageing population, better reporting, and a true increase in incidence. The exact cause of the rise in incidence remains speculative, but conforms to the Surveillance, Epidemiology and End Results (SEER) report [12]. Two-thirds of the patients presented with high-risk disease. Our present results are consistent with reports from Korea, China and Saudi Arabia, showing more men to have high-risk disease compared to patients with PCa in Western countries [5,11,13]. Ethnic variations in clinical stage at presentation in patients with PCa are well reported [13–15]. For example, minority populations in the USA present with more advanced symptoms and clinical stage of disease compared to the White Caucasian population [13–15]. These differences may be attributed to socioeconomic differences and access to healthcare rather than ethnic variation, as after corrections for all other factors, only socioeconomic status significantly affected the outcome of Black Americans compared to the White population [13–15]. Gleason score was found to be high in the minority ethnicities in the USA and was associated with higher mortality rates. The majority of Omani patients presented with high PSA levels and Gleason scores. Similar results were reported from Saudi Arabia [16]. These differences cannot be explained only by ethnic variability, but may reflect patients’ attitudes towards their health. Many previous reports clearly indicate that Omani patients present with advanced disease [8,9] despite there being a robust health system in place [17]. Our present data are consistent with the data reported from Saudi Arabia and Egypt, where patients present with high PSA levels and high Gleason scores [18,19]. Both these studies did not report clinical stage at presentation, treatment offered, or outcome of their patients and this makes our present study unique, as we report not only the clinicopathological features of localised PCa in an Arab population, but also the modality of treatment and survival outcomes.

PSA is widely used for screening and early detection of the PCa despite its lack of sensitivity and specificity [11]. Screening programmes do not exist in many countries in the region, and this may explain the advanced disease stage and high PSA levels at the time of diagnosis. On the other hand, a study from Kuwait showed that 11% of Kuwaitis were found to have PCa with a PSA level of >10 ng/mL [20]. High PSA levels have been reported in Arab and Asian men with benign prostate disease and a study from Saudi Arabia, revealed much lower rate of PCa in Saudi men compared to the Western population with similar PSA levels [18].

At our centre treatment is offered as per the NCCN guidelines and CAPRA risk stratification for patients with localised PCa. It is interesting that only a few patients accepted active surveillance as the treatment modality for very-low- or low-risk disease, mainly due to apprehension associated with the diagnosis of cancer. There has been a considerable difference in the choice of treatment options for men with localised PCa. In the past, more men, even with low-risk disease, were treated aggressively, while nearly half of patients with high-risk disease were treated with ADT [5,6]. Not a single guideline endorses primary ADT (PADT) as an option for patients with localised PCa, but there are reports of such an approach [6]. A study from Japan showed that PADT did not reduce the life span of older patients compared to the normal population and had low LHRH-associated adverse effects too. Contrary to that report, a similar approach had worse outcomes for American patients with PCa. Based on these data, the NCCN Asia Consensus Statement for Prostate Cancer supported PADT as a valid option for older Asian patients with PCa [6]. Our present data also suggest a similar practice treating older patients with localised PCa with PADT with various risk groups. Surprisingly, of the 10 patients who were treated with PADT, only one developed mCRPCa. However, a Korean study reported significantly poorer survival of patients treated with PADT compared to RP [6].

Recent data clearly suggest almost equal efficacy of PCa-related outcome for patients who were treated with RP or IMRT in combination with LHRH analogues, but with a difference of treatment duration and side-effects [4]. Almost an equal number of patients (34% and 36%) in our present cohort were treated with surgery or definitive IMRT along with variable periods of LHRH analogues, based on the risk stratification group.

At disease progression, treatment was offered based on the NCCN guidelines and consisted of docetaxel, abiraterone or enzalutamide according to the physician’s and patient’s choice and clinical characteristics of patients as suggested. The OS rates of our present cohort further validate our adherence of our clinical practices. The 5- and 10-year OS rate was 84% and 57%, respectively, which is similar or better than that reported from Korea and USA [11,13]. Among Asian countries the highest survival for patients with PCa has been reported from Japan, Korea and Singapore, while variable rates have been reported from China and lower OS has been reported from other countries, e.g. India, Iran, Thailand and Philippines. The differences in survival have been attributed to the Human Development Index (HDI) of these countries. Oman is among countries with a high HDI. The OS rates for patients with localised PCa in our present cohort were significantly better than countries with a lower HDI and lower than Japan and Singapore [7].

There are some limitations to our present study. The sample size was small, which might affect some of the reported outcomes. The data were collected from patients treated over a long period, extending from 2006 to 2017, and change in treatment recommendations may have affected the outcomes. However, during the past decade there has not been much change in the treatment, except for recent reports of upfront docetaxel in patients with high-risk localised PCa. Moreover, the present study is a single-centre study, hence does not provide complete country data, but as stated earlier half of the patients with cancer in Oman are treated at the SQUH, thus this might not affect the remaining data considerably. Besides that, the present study is a retrospective study and biases of retrospective studies are well-known in the literature. Nevertheless, this is the first report from this region and may provide a benchmark for subsequent studies from the region.

In conclusion, Omani patients with localised PCa present with a high PSA level and high Gleason score. It is still early to comment on the survival outcomes, as the median follow-up time of 43 months is considered a relatively short period to report outcomes of patients with localised PCa.

## Data Availability

The data that support the findings of this study are available from the corresponding author (Shiyam Kumar), upon reasonable request.

## Geographical information

Oman.
